# Optimizing a PCR protocol for *cpn*60-based microbiome profiling of samples variously contaminated with host genomic DNA

**DOI:** 10.1186/s13104-015-1170-4

**Published:** 2015-06-20

**Authors:** Lisa A Johnson, Bonnie Chaban, John C S Harding, Janet E Hill

**Affiliations:** Department of Veterinary Microbiology, Western College of Veterinary Medicine, University of Saskatchewan, Saskatoon, SK S7N 5B4 Canada; Department of Life Sciences, Imperial College London, London, SW7 2AZ UK; Department of Large Animal Clinical Sciences, Western College of Veterinary Medicine, University of Saskatchewan, Saskatoon, SK S7N 5B4 Canada

**Keywords:** cpn60, Microbiome profiling, Pig microbiome, PCR conditions, Gradient PCR, Pyrosequencing, Host microbiome

## Abstract

**Background:**

The current recommended protocol for chaperonin-60 (*cpn*60) universal target based microbiome profiling includes universal PCR of microbiome samples across an annealing temperature gradient to maximize the diversity of sequences amplified. However, the value of including this gradient approach has not been formally evaluated since the optimization of a modified universal PCR primer cocktail for *cpn*60 PCR. PCR conditions that maximize representation of the microbiome while minimizing PCR-associated distortion of the community structure, especially in samples containing large amounts of host genomic DNA are critical. The goal of this study was to measure the effects of PCR annealing temperature and the ratio of host to bacterial DNA on the outcome of microbiota analysis, using pig microbiota as a model environment.

**Findings:**

Six samples were chosen with an anticipated range of ratios of pig to bacterial genomic DNA, and universal *cpn*60 PCR amplification with an annealing temperature gradient was used to create libraries for pyrosequencing, resulting in 426,477 sequences from the six samples. The sequences obtained were classified as target (*cpn*60) or non-target based on the percent identity of their closest match to the cpnDB reference database, and target sequences were further processed to create microbiome profiles for each sample at each annealing temperature. Annealing temperature affected the amount of PCR product generated, with more product generated at higher temperatures. Samples containing proportionally more host genomic DNA yielded more non-target reads, especially at lower annealing temperatures. However, microbiome composition for each sample across the annealing temperature gradient remained consistent at both the phylum and operational taxonomic unit levels. Although some microbial sequences were detected at only one annealing temperature, these sequences accounted for a minority of the total microbiome.

**Conclusions:**

These results indicate that PCR annealing temperature does have an affect on *cpn*60 based microbiome profiles, but that most of the differences are due to differences in detection of low abundance sequences. Higher annealing temperatures resulted in larger amounts of PCR product and lower amounts of non-target sequence amplification, especially in samples containing proportionally large amounts of host DNA. Taken together these results provide important information to guide decisions about experimental design for *cpn*60 based microbiome studies.

**Electronic supplementary material:**

The online version of this article (doi:10.1186/s13104-015-1170-4) contains supplementary material, which is available to authorized users.

## Background

Complex microbial communities play a fundamental role in the health of animals, humans and the environment, and new opportunities to more fully characterize and understand these communities are available due to advances in sequencing technologies. The chaperonin-60 universal target (*cpn*60 UT, a 549–567 bp segment of the *cpn*60 gene) has been demonstrated to be a preferred barcode sequence for bacteria [1], and an especially useful target for high resolution microbiome profiling [2–10]. However, this approach, like any other that relies on PCR amplification of a target gene, distorts our view of the true community composition [11, 12].

One parameter of PCR that contributes to amplification bias is the annealing temperature. Previous work has indicated that performing the *cpn*60 UT PCR across a range of annealing temperatures and pooling the results improves the comprehensive assessment of microbial diversity of a sample [13]. The current recommended protocol involves 12 PCR reactions across an annealing temperature gradient for each sample that are subsequently pooled to create a sequencing library [14]. Including a full temperature gradient adds expense and logistical complexity to the protocol, and the importance of this procedure has not been re-evaluated since the development and application of a universal primer cocktail that improves representation of high G + C content organisms [15, 16], and coincident adoption of new generations of Taq polymerases and other PCR reagents.

Another parameter that may affect how a microbial community is amplified is the ratio of host to microbial genomic DNA. Large proportions of host genomic DNA can interfere with the amplification of *cpn*60 UT, resulting in non-target amplification. A previous study of the upper respiratory tract of pandemic H1N1 influenza patients demonstrated that samples containing large proportions of human DNA resulted in non-target amplification originating from the human genome during sequencing [3]. Although comprehensive microbiome profiles were generated, it was with additional, perhaps unnecessary cost due to greater sequencing depth requirements to ensure adequate coverage of the microbiome in the context of large amounts of host genomic DNA.

The goal of this study was to measure the effects of PCR annealing temperature and the ratio of host to bacterial DNA on the outcome of *cpn*60-based microbiota analysis using the recommended PCR primer cocktail, and pig microbiota as a model environment.

## Methods

### Sample collection and DNA extraction

Six samples were collected, five from different anatomical sites within a single pig, and one from an environmental soil sample. Samples were chosen to represent an anticipated range of pig to bacterial DNA ratios, from entirely pig to entirely bacterial. Samples were collected from a pig with congenital nasal obstruction, euthanized in an unrelated study designed and conducted in accordance with the Canadian Council for Animal Care and approved by the University of Saskatchewan Committee on Animal Care and Supply (Protocol #20130034). Samples collected included a section of brain tissue, a nasal swab, a stomach mucosal scraping, a colon mucosal scraping and feces (collected from rectum). Soil collected from the grounds of the University of Saskatchewan campus, was used as a pig genomic DNA free sample. All samples were stored at −20°C until DNA extraction.

Total genomic DNA extraction was performed on 0.2 g of material from the six samples using a previously described protocol that combines mechanical disruption and chemical extraction [17]. An extraction blank, consisting of ultrapure water, was carried through DNA extraction and sequencing. The DNA concentration of each sample extract was measured by spectrophotometry, and samples were diluted to a concentration of 10 ng/μl for PCR.

### Quantitative PCR (qPCR)

Quantification of bacterial and pig genomic DNA in each sample was performed by qPCR using the primers as described in Table 1. Estimation of bacterial content was obtained using primers targeting the V1–V3 region the 16S rRNA gene [18] in a qPCR assay described previously [3], while estimation of pig genomic DNA content was based on the quantification using the pig alpha actin gene.

**Table 1:** PCR primer sequences

Target	Primer	Sequence (5′–3′)^a^	Annealing temp (°C)	Reference
*cpn*60 UT	H279	GAIIIIGCIGGIGAYGGIACIACIAC	42, 48, 54, 60	[15]
	H280	YKIYKITCICCRAAICCIGGIGCYTT		
	H1612	GAIIIIGCIGGYGACGGYACSACSAC		
	H1613	CGRCGRTCRCCGAAGCCSGGIGCCTT		
16S rRNA gene	SRV3-1	CGGYCCAGACTCCTAC	62	[18]
	SRV3-2	TTACCGCGGCTGCTGGCAC		
Pig α-actin gene	JH0462	CCCAGAGCAAGCGAGGTATT	68	This study
	JH0463	GGGCCTCAGTGAGCAGAGTA		

^a^I = inosine, Y = G or T, K = G or T, R = A or G, S = G or C.

All qPCR reactions were performed in duplicate, including a no template control (NTC) and a standard curve, which was prepared with the target-containing plasmids at concentrations of 10^0^–10^7^ copies/reaction. Each reaction contained 1 × iQ SYBR Green Supermix (Bio-Rad, Mississauga, ON, Canada), 400 nM of both forward and reverse primers, and 2 μl of template DNA, in a final volume of 25 μl. All reactions used the following program: 95°C for 3 min, 40 cycles of 95°C for 15 s, annealing temperature (Table 1) for 15 s, 72°C for 15 s, and final extension at 72°C for 5 min. A dissociation curve was also run for 81 cycles at 0.5°C increments from 55 to 95°C. PCR was performed on a MyiQ thermocycler (Bio-Rad), and the data was analyzed using iQ5 Optical System Software.

### cpn60 universal target PCR and pyrosequencing

Amplification of the UT region of the *cpn*60 gene was performed using a primer cocktail consisting of a 1:3 molar ratio of primers H279/H280:H1612/H1613 [15] (Table 1). Primers were modified with the addition of multiplexing ID tags at the 5′ end. Each PCR reaction contained 1 × PCR reaction buffer, 2.5 mM MgCl_2_, 200 μM dNTP, 800 nM primer cocktail, 2.5 U Platinum Taq DNA Polymerase (Invitrogen, Burlington, ON, Canada) and 2 μl of template DNA, in a final volume of 50 μl. For each sample, four PCR reactions were performed in a thermocycler (Eppendorf Mastercycler) over an annealing temperature gradient with the following program: 94°C for 5 min, 40 cycles of 95°C for 30 s, annealing temperature (Table 1) for 30 s, and a final extension of 72°C for 2 min. A NTC and positive amplification control were included with each gradient. PCR products (5 μl) were resolved and visualized on a 1% agarose gel by ethidium bromide staining. Products were then purified by agarose gel extraction (QIAEX II gel extraction kit, Qiagen, Mississauga, ON, Canada) and suspended in TE buffer [10 mM Tris (pH 8), 1 mM EDTA]. The resulting 28 amplicon libraries were prepared and pooled in equimolar concentrations and sequenced using the Roche 454 GS Junior system as per manufacturer’s instructions.

### OTU assembly and analysis

Raw pyrosequencing data was processed by using the default on-rig procedures from 454/Roche. Filter-passing reads were used in the subsequent analyses for each of the pyrosequencing libraries. Sequence reads were de-multiplexed and processed to generate operational taxonomic units (OTU) with the microbial Profiling Using Metagenomic Assembly (mPUMA) pipeline [19] using Trinity for OTU assembly. Processing of sequence reads by mPUMA includes identification and removal of amplification primer sequences and identification of putative chimeras using the C3 chimera checker. Watered-BLAST [20] comparison to the cpnDB_nr reference database (version 20130321, downloaded from http://www.cpndb.ca) [21] was used to identify each OTU. Sequences identified as non-target were reference mapped (GS Reference Mapper, Roche, Bradford, CT, USA) to the pig genome (*Sus scrofa*, Genbank Accession AEMK01000000) to determine the amount of non-target amplification of pig genome origin. Coverage and Shannon diversity for each library was calculated using Mothur [22]. Principal coordinates analysis of jackknifed Bray–Curtis dissimilarity matrices was performed in QIIME [23].

## Results and discussion

### Genomic DNA extraction and quantification

Estimated copy numbers of pig genomes and bacterial genomes were determined by qPCR targeting the pig α-actin or bacterial 16S rRNA genes. Starting quantities were calculated based on interpolation using a standard curve of a ten-fold dilution series of plasmid containing the target sequence. For the α-actin PCR, a linear standard curve was obtained over a range of 10^2^–10^7^ copies per reaction (efficiency 105%, r^2^ = 0.996). For the 16S rRNA PCR, a linear standard curve was obtained over a range of 10^4^–10^7^ copies per reaction (efficiency 73%, r^2^ = 0.955). Based on the estimate copy numbers, ratios of pig and bacterial genomic DNA were 80:1 (brain), 0.8:1 (nasal), 2.3:1 (stomach), 1.3:1 (colon), 0.0004:1 (feces), and 0:100 (soil) (Table 2).

**Table 2:** Quantification of genomic DNA extracted from pig tissue and soil microbiomes

Sample	Gene copies per 20 ng DNA^a^		
	Pig α-actin	Bacterial 16S rRNA	Pig:bacteria DNA ratio
Brain	9.57 × 10^5^ ± 2.75 × 10^5^	1.20 × 10^4^ ± 1.79 × 10^3^	80:1
Nasal cavity	5.65 × 10^6^ ± 7.07 × 10^4^	6.77 × 10^6^ ± 6.91 × 10^5^	0.8:1
Stomach mucosa	4.76 × 10^6^ ± 1.23 × 10^6^	2.04 × 10^6^ ± 1.26 × 10^5^	2.3:1
Colon mucosa	8.21 × 10^6^ ± 2.58 × 10^3^	6.08 × 10^6^ ± 3.30 × 10^4^	1.3:1
Feces	1.09 × 10^5^ ± 6.21 × 10^3^	2.45 × 10^8^ ± 4.79 × 10^7^	0.0004:1
Soil	Not detected	3.81 × 10^5^ ± 2.82 × 10^5^	All bacterial

^a^Quantities reported are the average of duplicates ± standard deviation.

### cpn60 PCR and pyrosequencing

**Figure 1:**
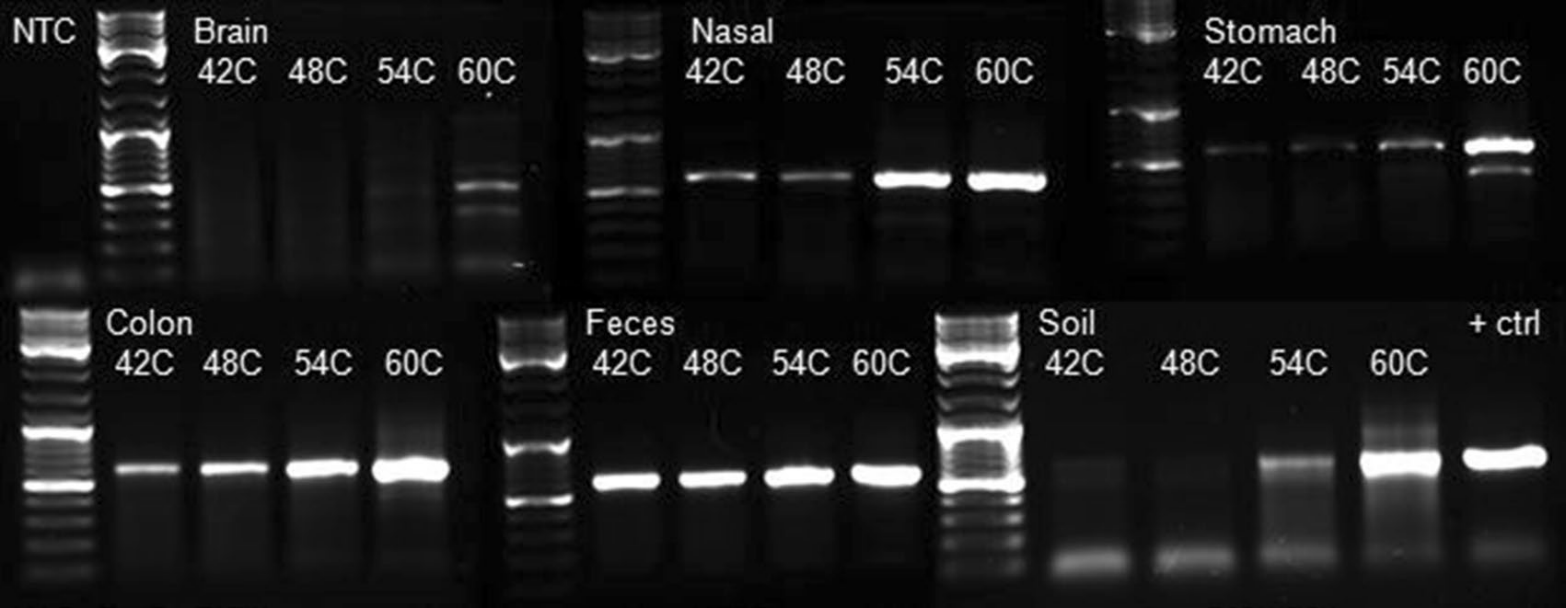
*cpn*60 PCR products from each sampling site at each PCR annealing temperature tested visualized on a 1% agarose gel. An equal volume of PCR product (5 µl) from each sampling site (brain, nasal, stomach, colon, feces, and soil) and each PCR annealing temperature gradient point tested (42, 48, 54 and 60°C) were visualized next to a ladder to determine amplification efficiency. Major bands on the DNA ladder indicate 500, 1,000 and 3,000 bp, while the *cpn*60 UT product is ~650 bp. *NTC* no template control, *+ctrl* positive control sample (genomic DNA from *Helicobacter canis*).

The *cpn*60 UT PCR products generated for each sample across the annealing temperature gradient were visualized on an agarose gel (Figure 1). The amount of PCR product produced varied by sample type and annealing temperature. For all sample types, the amount of PCR product generated increased with higher annealing temperatures, indicating that annealing temperature can have a dramatic effect on the amount of PCR product produced. The relationship between annealing temperature and PCR efficiency is well known [24]. Increasing hybridization stringency by elevating the annealing temperature reduces the occurrence of non-productive annealing events where primers anneal to non-target regions of the template. Although these interactions could lead to primer extension, the resulting products would be unlikely to participate in further amplification and could interfere with amplification of target regions by providing primer annealing sites.

Sequencing from the 28 libraries resulted in 426,477 high quality reads, with library sizes ranging from 1,707 to 81,128 reads (median 10,124). To facilitate comparison and reduce errors in interpretation due to unequal sampling depth [25], libraries were sub-sampled to the size of the smallest library of 1,707 reads. Coverage values for the subsampled pig derived amplicon libraries were >0.94. Coverage for the soil libraries ranged from 0.81 to 0.88, reflecting the greater diversity of these samples (see below). Reads were assembled into OTU and compared to the cpnDB reference database for assignment of nearest neighbour taxonomic labels to *cpn*60 (target) OTU, and to identify non-target sequences. OTU sequences were classified as target (*cpn*60) or non-target based on the percent identity of their closest database match. Our previous experience and analysis of *cpn*60 reference data in cpnDB has led the establishment of a cutoff of 55% identity for discrimination of *cpn*60 and non-target sequences. Additional file 1 shows the typical, bimodal distribution of percent identity values observed when amplicon libraries include non-target sequences. Detailed analysis of non-target OTU is described below. OTU identified as *cpn*60 sequences (1164/2164 OTU assembled) corresponded to 480 nearest neighbour species: 380 OTU detected in soil, 104 in colon, 103 in feces, 52 in stomach, 23 in nasal swab, and 36 in brain. A summary of the nearest neighbour species detected in each sample and the number of sequence reads associated with each is given in Additional file 2.

### Target and non-target amplification

**Figure 2:**
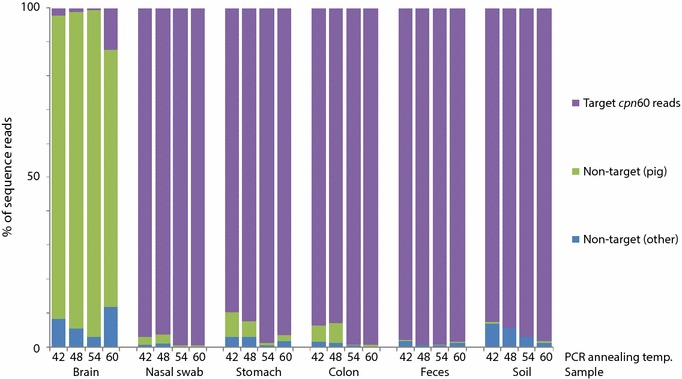
Proportion of each pyrosequencing library that was composed of target (*cpn*60) reads and non-target (of pig or other origin) reads. Libraries are labeled by sample site and PCR annealing temperature.

OTU with <55% identity to the closest match in the cpnDB reference database were considered non-target and removed from microbiota analysis. Sequence reads contributing to these non-target OTU were screened against the pig genome to identify non-specific amplification products of pig origin. Any remaining sequences, likely of microbial origin, were assigned to the category non-target (other origin). The proportions of *cpn*60 target and non-target (pig and other origin) sequence reads in each library are shown in Figure 2. The average target:non-target ratio across the four annealing temperature libraries for each sample were 4:96 (brain), 98:2 (nasal), 94:6 (stomach), 96:4 (colon), 99:1 (feces) and 96:4 (soil). Furthermore, samples containing appreciable proportions of pig genomic DNA (brain, nasal swab, stomach and colon mucosal scrapings) yielded more non-target reads corresponding to the pig genome, especially at lower annealing temperatures. For example, the brain sample, which was estimated to contain 99% pig genomic DNA, resulted in 89% of the total reads corresponding to non-target pig genome amplification. Alternatively, the nasal and stomach samples, which contained 45 and 70% pig genomic DNA respectively, resulted in 1 and 4% of the total reads to correspond to the pig genome.

The profiles corresponding to the brain sample libraries contained from 0.8 to 12.3% bacterial *cpn*60 sequences (269 reads total) (Figure 2). While some of these sequence reads may originate from the brain tissue, trace contamination was detected in the extraction negative and some of these sequences were also detected in the brain libraries. Thus, the detection of microbial sequences the brain libraries was at least partially due to contamination of the sample during collection at necropsy, and/or during laboratory processing. Brain microbial reads were not analyzed further.

There was an observed trend of greater proportions of non-target amplification at lower annealing temperatures, especially in samples containing large proportions of host DNA (Figure 2). This finding is consistent with a previous *cpn*60 microbiome study where non-target amplification from samples of the human upper respiratory tract, containing an order of magnitude more human DNA than bacterial DNA, accounted for up to 85% of the data generated [5].

### Effect of annealing temperature on phylum level profiles

**Figure 3:**
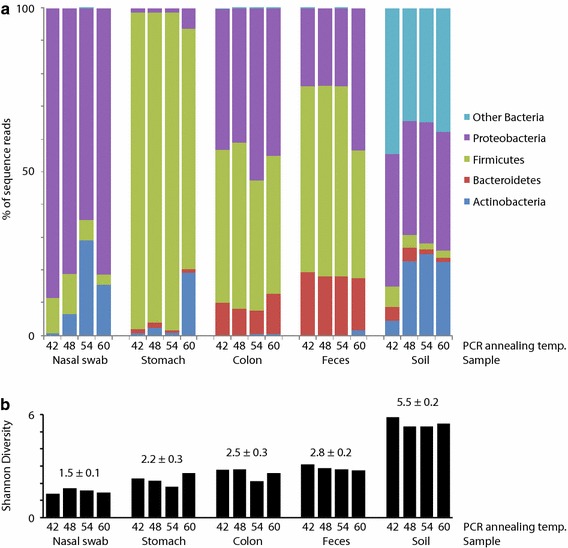
Composition and diversity of PCR product libraries. Phylum level composition (**a**) and Shannon diversity values (**b**) for each library are shown. Mean Shannon diversity value ± standard deviation is shown above the *bars* representing individual PCR product libraries.

*cpn*60 OTU were used to generate phylum level microbiome profiles, which were generally consistent within body site, regardless of annealing temperature (Figure 3). The nasal passage libraries were dominated by Proteobacteria (average of 4 annealing temperature libraries, 79%), Actinobacteria (13%) and Firmicutes (8%), similar to previous descriptions of the pig tonsil microbiome [26]. The stomach libraries contained mostly Firmicutes (91%), with some Actinobacteria (6%) and Proteobacteria (3%), consistent with the most abundant phyla identified in the stomach microbiomes of horses [27]. The colon and feces libraries were dominated by Firmicutes (45 and 53%, respectively), Proteobacteria (46 and 29%), and Bacteriodetes (9 and 18%) as expected based on previous descriptions of these environments [28, 29]. The soil libraries included Proteobacteria (37%), Actinobacteria (19%), Bacteriodetes (3%), and Firmicutes (3%), with 38% of the reads belonging to other phyla. Diversity was lowest in the nasal swab libraries (Shannon diversity 1.5 ± 0.1) and highest in the soil libraries (5.5 ± 0.2), and no consistent relationship of annealing temperature and library diversity was observed (Figure 3b).

### Effect of annealing temperature on species level profiles

**Figure 4:**
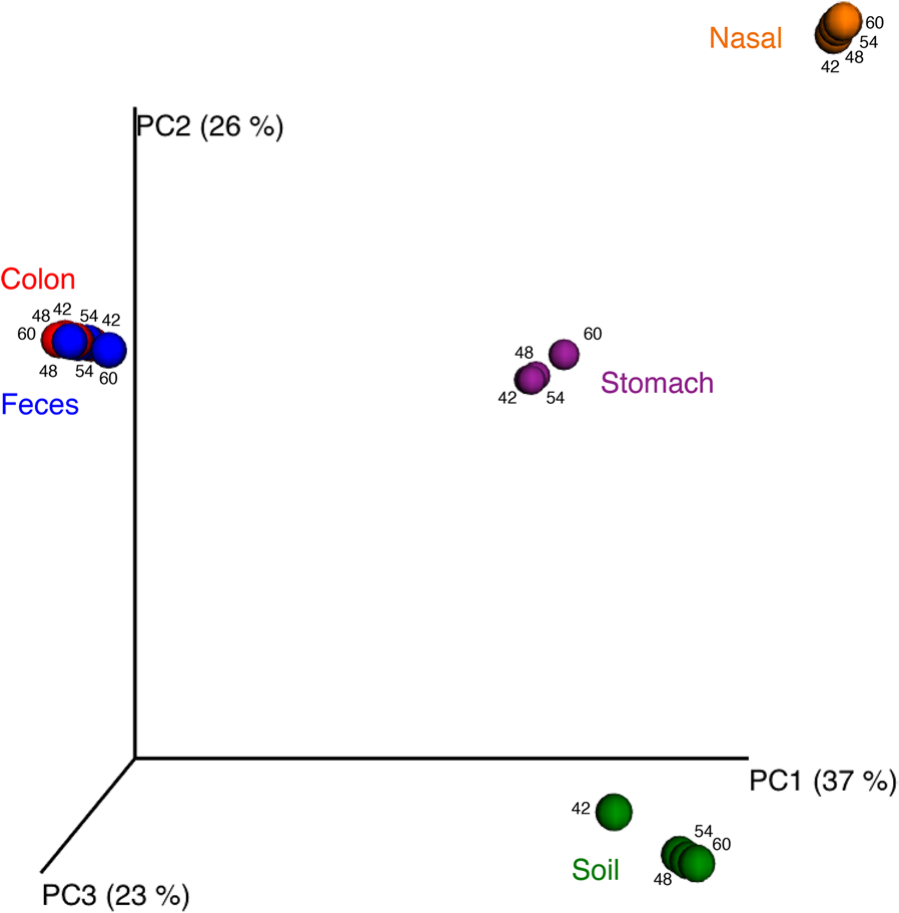
Principal coordinates analysis based on Bray–Curtis dissimilarity values. The analysis was based on the average pairwise dissimilarities from 100 jackknifed data sets at a sampling depth of 1,000 reads per sample. Sample of origin and annealing temperature for each microbiome are indicated.

To expand on the phylum level comparisons and make a more detailed comparison of the microbial profiles produced across the annealing temperature gradient, we investigated whether different taxa were detected at different annealing temperatures. OTU sequences with the same nearest neighbour in cpnDB were combined as nearest neighbour “species” and their abundances combined accordingly for this analysis (Additional file 2). At this level, the overall similarity between microbiome profiles generated at different annealing temperatures is also apparent. Figure 4 shows the results of principal coordinates analysis based on Bray–Curtis dissimilarity values for species level microbiome profiles of all samples and annealing temperatures. Microbiome profiles cluster according to tissue or material of origin, with feces and colon overlapping.

**Figure 5:**
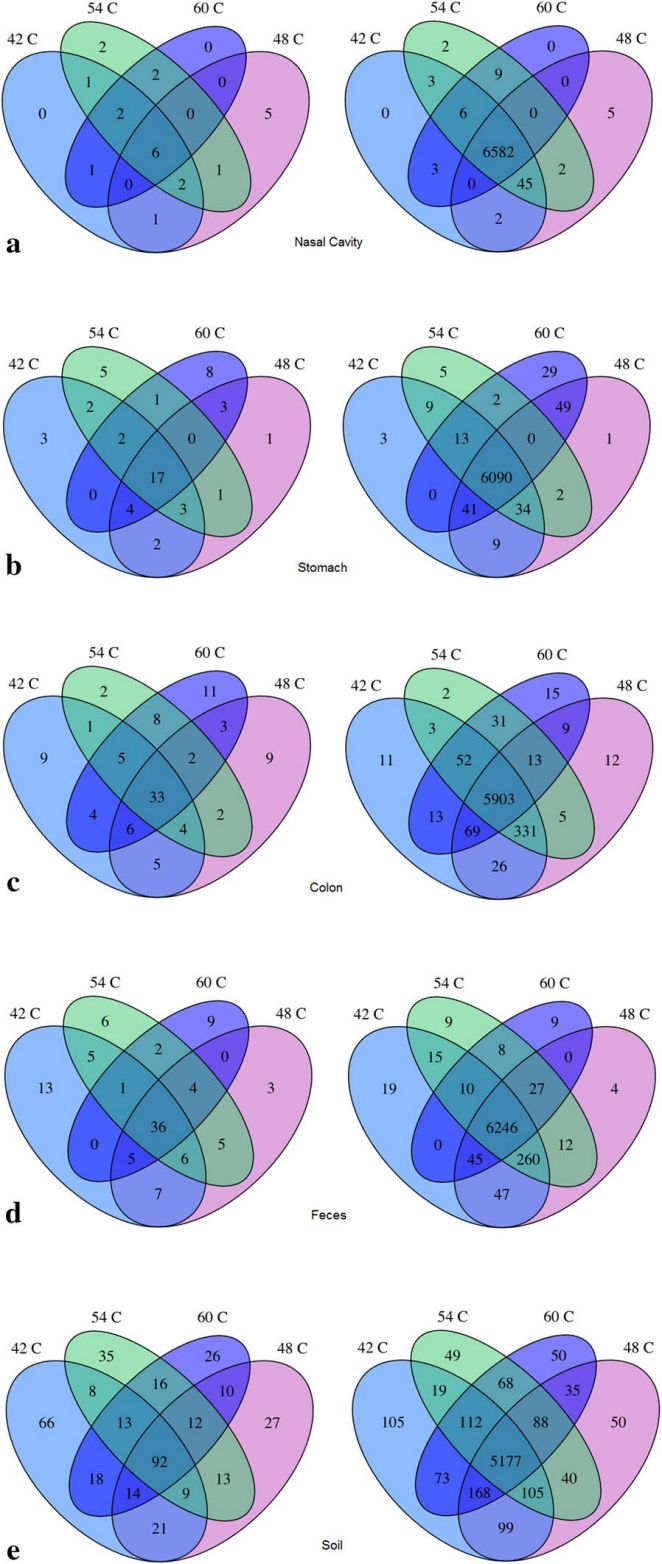
Number of nearest neighbour species (*left*) and sequence reads associated with OTU (*right*) detected at each annealing temperature for samples of **a** nasal cavity, **b** stomach, **c** colon, **d** feces and **e** soil.

To further understand the relationship between OTU abundance and prevalence in the different annealing temperature libraries we examined the distribution of individual OTU sequences. Figure 5 shows the number of nearest neighbour species (left side) and the number of sequence reads associated with OTU (right side) detected at specific annealing temperatures for each sample type. For each sample type, the total number of sequence reads was obtained by combining the subsampled libraries for each annealing temperature. The proportion of total species that were detected at all four annealing temperatures ranged from 24.2% (92/380) for soil to 35.3% (36/102) for feces (Figure 5, left). However, when abundance was considered, it was apparent that the majority of sequence reads were associated with OTU that were detected at all annealing temperatures (Figure 5, right). For all samples, OTU sequences detected at all annealing temperatures accounted for a minimum of 83.0% of reads. For example, although 8 species were detected only in the 60°C stomach library, these OTU accounted for only 1.7% of the data. Overall, for the pig microbiomes analyzed, from 86.5% (colon) to 96.4% (nasal swab) of the sequence reads were associated with OTU detected at all annealing temperatures. Interestingly, the proportion of sequence reads associated with OTU detected at all annealing temperatures was noticeably lower for the soil library where these common OTU accounted for only 75.8% of the sequence data, which reflects the greater diversity and more even frequency distribution of OTU in this microbiome relative to the pig associated communities.

Nearest neighbour species could be detected at specific annealing temperatures because they are preferentially amplified at those annealing temperatures, however this was not addressed in our study. An alternative explanation is that these OTU were simply so rare in the starting sample that they were only detected sporadically at the sequencing depth used. For example, in the nasal libraries, 2/23 OTU were detected at 42, 54 and 60°C but not at 48°C (Figure 5a, left). However, these OTU account for only 6/6,828 sequence reads and so it seems plausible that additional sequencing depth in the 48°C library would result in their detection. Indeed, it was observed that for samples where additional sequence reads were available, addition of more sequences into the analysis resulted in detection of more of the rare OTU in more annealing temperature libraries, and thus fewer nearest neighbours being detected only at one annealing temperature (data not shown).

## Conclusion

The universal *cpn*60 PCR annealing temperature had effects on the amount of PCR product produced and the microbiota profiles produced at both phylum and OTU levels. However, the differences observed in profiles were largely due to low abundance sequences, especially in the animal associated microbiomes examined. The amount of non-target amplification was somewhat greater at lower annealing temperatures, but was most prominent for samples containing large proportions of host genomic DNA. OTU uniquely detected at certain annealing temperatures were rare, and accounted for a small proportion of microbiota profiles, although this finding was affected by sampling depth. Taken together, these results indicate that the logistically complex and expensive process of including a full temperature gradient in *cpn*60 amplicon library production may not be necessary, depending on the goals of the experiment. In cases where samples are known to contain large amounts of host DNA, amplification at the maximum annealing temperature feasible will minimize non-target amplification. However, practitioners of *cpn*60 based microbiome profiling should also consider that if detection of rare OTU is critical to the experimental goals, an annealing temperature gradient may still prove useful to increase the likelihood of capturing of all of the microbial diversity present in the environment of interest.

### Availability of supporting data

The data set supporting the results of this article is available in the NCBI SRA repository, and is associated with BioProject accession PRJNA260274, http://www.ncbi.nlm.nih.gov/bioproject/PRJNA200951.

## Additional files

Additional File 1: Title of data: Distribution of percent identities of *cpn*60 OTU sequences to their nearest neighbour in the cpnDB reference database. Description of data: The lower distribution (OTU with <55% identity) contains non-*cpn*60 sequences, largely pig genomic DNA sequences, which were subsequently mapped on to the pig genome for confirmation of their origin. The majority of pig genomic DNA sequences were assembled from the brain tissue sample, which also had the highest ratio of host:bacteria genomic DNA of any of the samples in the study. Manual inspection of OTU sequences with <55% identity but that did not map on to the pig genome indicated that many of them were of bacterial origin (bacterial, non-*cpn*60 sequences). Additional File 2: Title of data: Nearest neighbour species detection frequencies. Description of data: The number of sequence reads associated with each nearest neighbour species detected in each sample library are shown.

## Abbreviations

OTU: operational taxonomic unit
*cpn*60: chaperonin-60
UT: universal target
NTC: no template control
mPUMA: microbial Profiling Using Metagenomic Assembly
