# Supplementary material for: Optimizing a PCR protocol for cpn60-based microbiome profiling of samples variously contaminated with host genomic DNA
Source: BMC Res Notes. 2015 Jun 20;8:253. doi: 10.1186/s13104-015-1170-4 (PMC4475309; doi:10.1186/s13104-015-1170-4)
Supplement: Additional File 1: — Title of data: Distribution of percent identities of cpn60 OTU sequences to their nearest neighbour in the cpnDB reference database. Description of data: The lower distribution (OTU with <55% identity) contains non-cpn60 sequences, largely pig genomic DNA sequences, which were subsequently mapped on to the pig genome for confirmation of their origin. The majority of pig genomic DNA sequences were assembled from the brain tissue sample, which also had the highest ratio of host:bacteria genomic DNA of any of the samples in the study. Manual inspection of OTU sequences with <55% identity but that did not map on to the pig genome indicated that many of them were of bacterial origin (bacterial, non-cpn60 sequences). [file 13104_2015_1170_MOESM1_ESM.pdf]

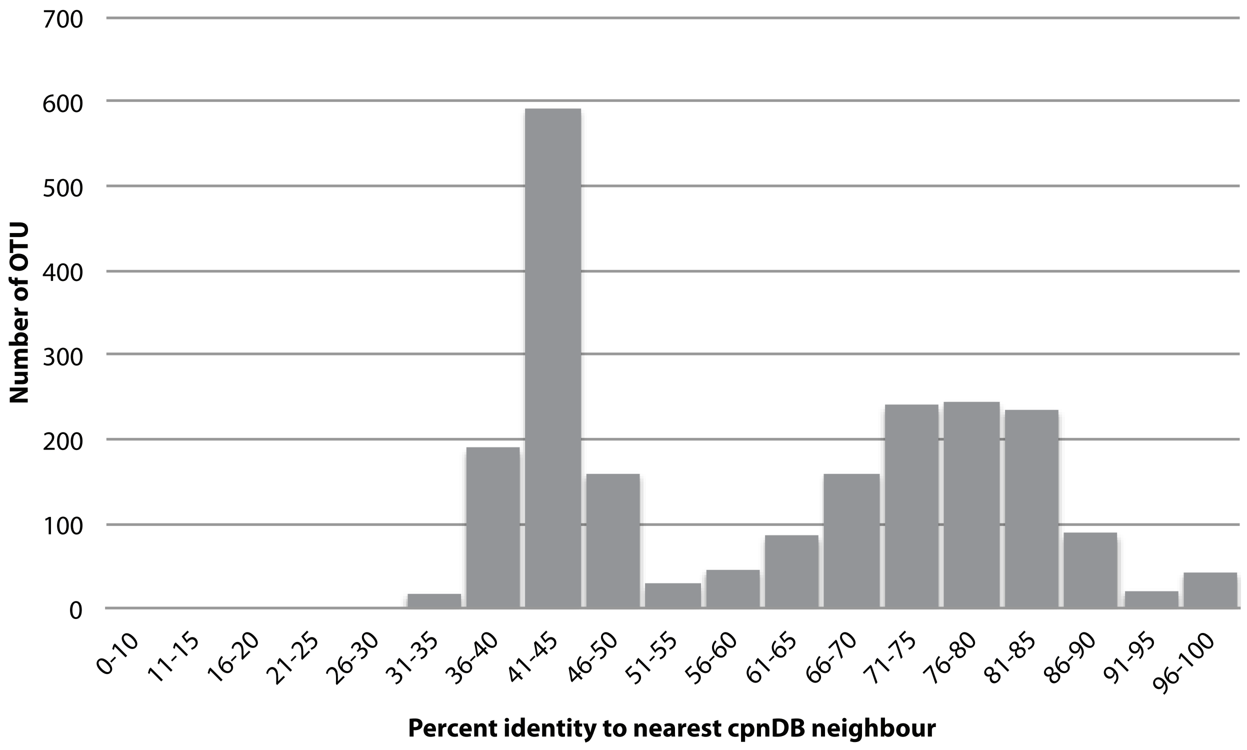

Additional File 1. Distribution of percent identity values for study OTU to their best match (nearest neighbour) in the cpnDB reference database. The lower distribution (OTU with <55% identity) contains non-cpn60 sequences, largely pig genomic DNA sequences, which were subsequently mapped on to the pig genome for confirmation of their origin. The majority of pig genomic DNA sequences were assembled from the brain tissue sample, which also had the highest ratio of host:bacteria genomic DNA of any of the samples in the study. Manual inspection of OTU sequences with <55% identity but that did not map on to the pig genome indicated that many of them were of bacterial origin (bacterial, non-cpn60 sequences).
